# Henry S. Kaplan Distinguished Scientist Award

**DOI:** 10.1093/jrr/rrt148

**Published:** 2014-01

The International Association for Radiation Research established in 1985 the Henry S. Kaplan Distinguished Scientist Award. The award, which is presented every four years at the International Congress of Radiation Research, consists of a medal, a certificate and a cash prize. A plenary session is designated for the award ceremony, including a lecture by the awardee. Previous recipients of this award were Professors Mortimer M. Elkind (1987), H. Rodney Withers (1991), Gerald Edward Adams (1995), John B. Little (1999), Eric J. Hall (2003), J. Martin Brown (2007) and P. Richard Hill (2011).

The purpose of the award is to honour outstanding contributions to the field of radiation research. Any scientist who has made superior contributions to the field through investigations in physics, chemistry, biology or medicine is eligible.

Nominations may be submitted either by an individual or by a society. Each nomination should include a current curriculum vitae, bibliography and a brief *(e.g.* 3-page) narrative describing the accomplishments of the nominee. Additional letters of support, up to a maximum of two, may be included with the nomination papers.

Nominations for the 2015 award are now invited, and should be submitted by 1^st^ May 2014 to Professor Gianfranco Grossi, Secretary-Treasurer IARR, Department of Physics, University Federico II, Complesso Universitario Monte Sant'Angelo, Via Cinthia, I-80126 Napoli, Italy (gianfranco.grossi@unina.it). All nomination packages will be forwarded to the Selection Committee, comprising Professors Marco Durante, Mary Helen Barcellos-Hoff and Ohtsura Niwa. The recipient of the Kaplan award will be notified before the end of October 2014.

